# Editorial: Cerebral vessel extraction—from image acquisition to machine learning

**DOI:** 10.3389/fnins.2022.972389

**Published:** 2022-07-13

**Authors:** Binjie Qin, Sung-Liang Chen, Peng Miao, Zhongzhao Teng

**Affiliations:** ^1^School of Biomedical Engineering, Shanghai Jiao Tong University, Shanghai, China; ^2^University of Michigan-Shanghai Jiao Tong University Joint Institute, Shanghai Jiao Tong University, Shanghai, China; ^3^Department of Radiology, University of Cambridge, Cambridge, United Kingdom

**Keywords:** cerebral vessel extraction, biomedial image analysis, image accquisition, machine learning, deep learning, neuroimaging, neurovascular complex, brain vessels

The brain is the most energetically demanding organ and relies on a continuous supply of nutrients and oxygen from the blood. To maintain energy supply and remove unwanted proteins and metabolites continuously, the cerebrovascular function of the brain relies on the normal functioning of the macrocirculation in cerebral arteries and the microcirculation in cerebral microvessels including capillaries (Hartmann et al., 2021; Schaeffer and Iadecola, 2021). Due to the hemodynamic response to neural activation facilitating the understanding of brain function and pathologies, extracting cerebral vessel networks for the measurement of vascular dynamics has proved to be very important for the early diagnosis of critical cerebrovascular and neurological disorders. During the past 20 years, new neuroimaging technologies including various optical imaging methods have been developed and validated in the basic neuroscience studies, while clinical contrast-enhanced imaging modalities including X-ray, CT, MRI and ultrasound have also advanced in cerebral vessel extraction to provide more precise and useful diagnostic information. Machine learning for big imaging data is enabling engineers to precisely extract the cerebral vessel networks for unveiling previously unknown information about neurovascular coupling at a range of spatiotemporal scales. In this special issue, five original articles describe state-of-the-art advances in various brain vessel image acquisition, image reconstruction and analysis, as well as machine learning technologies and, bringing together traditional and new imaging modalities into the basic research, preclinical studies and clinical applications.

The first study by Chen et al. has answered the question of whether the structural information of brain vessels can be extracted from the functional information, e.g., blood flow. They demonstrate that the laser speckle contrast imaging can obtain the blood flow of cerebral vessels in real time by reflecting the functional information about blood supply and tissue perfusion in the brain. In this work, the authors propose a deep learning-based method to rapidly segment cerebral vessels from contrast images containing the blood flow information. This method will help to improve the application of laser speckle contrast imaging in brain surgery.

Identification of symptomatic carotid artery plaque may be clinically valuable in recognizing vulnerable and high-risk carotid atherosclerosis, providing important information for treatment. Yang et al. report a clinical study of 90 patients comparing symptomatic and asymptomatic carotid plaque. To discriminate symptomatic carotid artery plaque, the authors used three factors including high-density lipoprotein cholesterol (HDL-C), angiographic irregular plaque, and white thrombus detected by optical coherence tomography (OCT) imaging using logistic regression. They achieved a satisfactory result with 59.4% sensitivity and 84.5% specificity. The developed three-item scale shows promise to determine symptomatic carotid artery plaque. Future large-scale studies to validate the three-item scale, and further refine the model to improve sensitivity and specificity are clearly warranted.

Currently, studying blood flow in the capillary network to understand how microvascular flow is regulated in the brain in health and disease is still a challenging problem. Stefan et al. have presented a deep learning and simulation-based method to measure cortical capillary red blood cell (RBC) flux in a high-throughput way using OCT as input data with simultaneously acquired two-photon excitation fluorescence microscopy acting as the ground truth of training data. The training data are learned to uncover the distribution of parameters governing the height, width, and inter-peak time of peaks in OCT intensity associated with the passage of RBCs. This can simulate thousands of time-series examples for different flux values and signal-to-noise ratios to train a 1D convolutional neural network (CNN). The trained CNN is applied to 4D data of the murine cortical vasculature to estimate RBC flux across the entire network of hundreds of capillaries. Repeating this prediction and averaging for every vessel in a capillary network produced a map of RBC fluxes that allow for the study of capillary network dynamics with greatly improved statistical power.

Radiotherapy is effective treatment strategy for head and neck cancer, however may induce cerebrovascular stenosis or occlusion during the follow-up. The mechanism of radiation-induced carotid stenosis (RICS) has been underdetermined. Further, carotid artery stenting is performed in patients with RICS to reduce the risk of stroke. Hence, studies to elucidate the mechanism of RICS are needed. Xu et al. recently studied five patients with RICS to evaluate the morphological features of RICS and stent-vessel interactions by intravascular OCT. They found that the morphological features of RICS were heterogeneous by OCT evaluation upon the carotid stent implantation, and one patient developed in-stent neointimal hyperplasia on follow-up OCT examination. The preliminary study demonstrates that OCT can be applied to reveal the features of RICS. Future studies on RICS by OCT will shed light on the understanding of RICS, thus improving its prognosis and treatment.

Although optical neuroimaging techniques including laser speckle contrast imaging, two-photon microscopy, and OCT have been widely used to measure blood hemodynamics in the brain vessels such as red blood cell velocity (RBCv) and/or cerebral blood flow (CBF), they are mostly restricted in brain surface for a limited coverage of a few individual cortical vessels. Due to the advent of ultrafast imaging at thousands of frames per second vastly enhancing slow blood flow sensitivity in smaller vessels, functional ultrasound imaging (Deffieux et al., 2021; de Paz and Macé, 2021) in recent study by Brunner et al. has been exploited to extract reliable RBCv information without the need of contrast agent or special acquisition requirement. With an angiographic scan of rat brain, the blood Doppler signal was filtered from slower movements or tissue part through 20Hz high-pass filter to be segmented as directional cortical microvessels with enough computed total signal intensity, which is split as positive and negative frequencies for the different flow directions in each angiographic image position. A sliding window Fourier transform of the segmented Doppler signal correctly quantified the Doppler spectrum in the blood flow direction for extracting the average spectrum and average RBCv in individual vessels via a simplified model of laminar flow and standard formula that links the velocity with the Doppler frequency. Then, the authors provided quantitative hemodynamic measurements of the absolute RBCv and relative CBF changes of individual cortical vessels under resting-state and evoked conditions. Hopefully, the functional ultrasound imaging modality will act as part of the toolkit for studying the neurovascular coupling at the brain-wide scale and new insights into neurological diseases.

Being represented from the different aspects of cerebral vessel extraction in the above studies, neurovascular complex (Schaeffer and Iadecola, 2021) is a recently emerged novel concept of not only investigating how neuronal signals regulate nearby microvessels to support the metabolic needs of the brain but also considering the cellular, molecular and functional diversity of the cerebrovascular tree and propagation of systemic vasoactive signals. It is suggested that the neurovascular complex comprises heterogeneous vascular modules regulated by factors intrinsic and extrinsic to the brain through segment-specific heterogeneity mechanisms. In machine learning for clinical application, the segmental heterogeneity of the vascular network also can be accurately and efficiently extracted to restore the diverse intensity and geometry profiles of heterogeneous vessels against complex interferences and dynamic backgrounds via model-based deep learning of X-ray coronary angiography (Qin et al., 2022). These advances in noninvasive biomedical imaging and machine learning of vascular tree heterogeneity as well as assessment of cerebrovascular function will provide the unique opportunity to examine the integrated regulation of the neurovascular complex in humans in health and disease and illuminate the functional interaction between extracranial and intracranial vessels and the effect of systemic factors, providing insight into the neurovascular coupling of complex behaviors and their alterations in disease for the diagnosis and treatment value.

## Author contributions

All authors contributed to conceptual design, writing, and approval of the manuscript.

## Funding

This study was partially supported by the Science and Technology Commission of Shanghai Municipality (19dz1200500 and 19411951507), the National Natural Science Foundation of China (61271320 and 61775134), and Interdisciplinary Program of Shanghai Jiao Tong University (ZH2018ZDA19, YG2021QN122, and YG2021QN99).

## Conflict of interest

The authors declare that the research was conducted in the absence of any commercial or financial relationships that could be construed as a potential conflict of interest.

## Publisher's note

All claims expressed in this article are solely those of the authors and do not necessarily represent those of their affiliated organizations, or those of the publisher, the editors and the reviewers. Any product that may be evaluated in this article, or claim that may be made by its manufacturer, is not guaranteed or endorsed by the publisher.
